# Intracellular pH Control by Membrane Transport in Mammalian Cells. Insights Into the Selective Advantages of Functional Redundancy

**DOI:** 10.3389/fmolb.2022.825028

**Published:** 2022-02-18

**Authors:** Denis Doyen, Mallorie Poët, Gisèle Jarretou, Didier F. Pisani, Michel Tauc, Marc Cougnon, Mederic Argentina, Yann Bouret, Laurent Counillon

**Affiliations:** ^1^ Université Côte d’Azur, CNRS, Laboratoire de Physiomédecine Moléculaire, Nice, France; ^2^ Laboratories of Excellence Ion Channel Science and Therapeutics, Nice, France; ^3^ Centre Hospitalier Universitaire de Nice, Service de Médecine Intensive Réanimation, Hôpital Archet 1, Nice, France; ^4^ Université Côte d’Azur, CNRS, Institut de Physique de Nice, INPHYNI, Nice, France

**Keywords:** pH regulation, pH measurements, transmembrane transport, mathematical modelling, functional redundancy, natural selection

## Abstract

Intracellular pH is a vital parameter that is maintained close to neutrality in all mammalian cells and tissues and acidic in most intracellular compartments. After presenting the main techniques used for intracellular an vesicular pH measurements we will briefly recall the main molecular mechanisms that affect and regulate intracellular pH. Following this we will discuss the large functional redundancy found in the transporters of H^+^ or acid-base equivalents. For this purpose, we will use mathematical modeling to simulate cellular response to persistent and/or transient acidification, in the presence of different transporters, single or in combination. We will also test the presence or absence of intracellular buffering. This latter section will highlight how modeling can yield fundamental insight into deep biological questions such as the utility of functional redundancy in natural selection.

## 1 Introduction

Temperature, pH and mechanical forces are fundamental physical parameters that affect living cells in all phyla. While cell mechanics have recently received a regain of attention, pH and temperature are often regarded as more trivial by non-specialists, possibly because they are easy to set and measure in everyday life, which in contrast is not the case at the cellular and subcellular scales. Also of note both pH and temperature have a vast biological impact from the tiniest molecular mechanisms to the planetary ecosystem. While recent publications show that temperature of biochemical mechanisms is far from being simple to measure and should receive more attention (Chretien et al., 2018; Lane, 2018); this review will deal with several aspects pertaining to pH regulation. As the cologarithm of free H^+^ concentration, pH quantifies the abundance of the smallest cation in the Universe, namely a proton, which is a hydrogen atom stripped of its electron. In the cytosol or in intracellular compartments pH is both one of the most controlled and one of the most challenging to control parameter. The reasons for this situation are at least threefold: 1) many reactions involve the fast release or consumption of protons an/or acid base equivalents, 2) cells are not simply chemical bags and use charge gradients across membranes to generate and store free energy. Those can directly be proton gradients such as in mitochondria, or indirectly provide and electro-osmotic driving force for protons, such as plasma membranes or in those of intracellular compartments, 3) the surface charge of macromolecules is dictated by their protonation state, which in turn depends on the availability of free H^+^ ions, namely on pH. The later reason is of utmost importance because interactions between molecules are built through electrostatics that are dictated by surface charge. In fine, pH, its gradients and dynamics dictate the space and time of macromolecular interactions.

In this context, evolution has selected an array of molecular mechanisms that address the challenge of regulating their pH by transporting acid-base equivalents across membranes. Those comprise pumps, leaks, and secondary transporters that can either translocate H^+^, bicarbonate or other buffers such as monocarboxylates.

In the last decades several colleagues have written excellent and comprehensive reviews on the various aspects of pH regulation in normal and pathological conditions [see for example (Casey et al., 2010; Damaghi et al., 2013; Parker and Boron, 2013; Rolver and Pedersen, 2021)]. In addition, this special issue, the present article is part of, also contains a set of articles covering several aspects of the field. Hence the aim of the present article is instead to provide in a first part a short focus on the methods of intracellular pH measurements and on the physical and chemical principles that govern intracellular pH regulation. In a second part, we will then link those two sections by illustrating how mathematical modeling can address the questions raised by the astonishing multiplicity of pH regulatory membrane transporters.

## 2 Measuring Cytosolic pH and the pH of Intracellular Compartments

Accurate determination of intracellular pH is of upmost importance for understanding the mechanisms that regulate this parameter. This requires not only to measure the steady state pH but also the dynamics of pH changes in various conditions. Because of these constraints pH measurements are challenging as they must be as least invasive as possible for cells while having the best spatiotemporal resolution as possible. This detracts from classical biochemical dosage techniques that usually lead to cell disruption or from whole cell patch clamp technique in which the intracellular contents are very rapidly dialyzed into the pipettes. Here also great reviews, that we cannot all cite, have been written on the different methods used to measure steady-state and pH variations both in cytosol and in organelles [see for example (Loiselle and Casey, 2010)].

Different systems have been used, from microelectrodes to the measurement of the diffusion of radiolabeled weak acids (Sardet et al., 1990) or electron microscopical quantification of immunoreactive weak bases in intracellular compartments (Anderson et al., 1984), but the most versatile technique, both for cytosolic and organelle pH measurements, is the use of pH sensitive fluorescent probes, whose absorption and/or emission spectra depend on their protonation state. In this case pH measurements just turn to be fluorescence spectroscopy measurements. Different setups can be used, from videomicroscopes equipped with camera and illumination systems (Milosavljevic et al., 2015) to fluorimeters or microplate readers equipped with fluorescence. Using fluorescence, pH values can be in principle deduced from a modified Henderson-Hasselbalch equation adapted to fluorescence, or fluorescence ratio when ratiometric probes are used. The advantage of the latter is that they offer internal correction for possible photobleaching or probe leakage. pH calibration is in general mandatory because the optical setup and cellular context can slightly modify the probes spectral properties or their pK_a_ values. For cytosolic pH this is achieved using Nigericin, a K^+^/H^+^ ionophore that enables to set precisely intracellular pH, using the fact that extracellular pH equals intracellular pH when extracellular and intracellular potassium concentrations are set at equilibrium. The pH of intracellular compartments requires more sophisticated calibration procedures because organellar lumen must be equilibrated with the cytosol that must be equilibrated with the extracellular medium. Those can use weak acid diffusion such as the null method (Eisner et al., 1989), or use combination of K^+^-H^+^ and Na^+^-H^+^ ionophores such as Nigericin and Monensin respectively (Milosavljevic et al., 2014).

### 2.1 Soluble Probe

Based on the previous considerations, a wide variety of fluorescent dyes whose fluorescence depends on their protonation, and therefore pH, exists and the reader can refer to comprehensive reviews such as (Han and Burgess, 2010). Of course, for optimal sensitivity they must be able to penetrate cells easily and then be retained in their cytosol or compartments. For this, many pH indicators are built as cleavable esters. Furthermore, their pK_as_ must be in the range of the pH values to be measured. Hence, archetypal probes with a pK_a_ nearby seven have been widely used for cytosolic pH. Those are fluorescein acetoxymethyl esters (e.g. BCECF); benzoxhantene dyes (e.g. SNARF), or cyanine-derived dyes.

To measure the pH of endocytic compartments soluble pH-dependent fluorophores can be coupled to molecules that can be endocytosed such as Dextran or transferrin. Because fluorescein has a pK_a_ of 6.3, such a strategy will work for early endosomes (Teter et al., 1998), but its use is limited in more acidic compartments such as late endosomes and lysosomes. This can be slightly improved by thiosocianate derivatives that will slightly reduce fluorescein pK_a_ (Ohkuma and Poole, 1978) or more strongly by substituting several hydrogen atoms by fluor atoms and carboxylic groups, leading to Oregon Green that has a pK_a_ around 4.7 (Saric et al., 2017). Non-fluorescein molecules with low pK_as_ are also in use, such as LysoSensor, (Lin et al., 2001), or Boron dipyrromethene (BODIPY) derivatives (Xia et al., 2019). Acridine orange derivatives, that accumulate in acidic organelles when protonated and fluorescent have been also used (Moriyama et al., 1982). Here also ratiometric approaches that can be used by coupling the probe to a pH stable GFP derivative (mCherry) are a great advantage (Saric et al., 2017). Pertaining to technical drawback to pay attention to is that, as the volume of these compartments is very tiny compared to cytosol, the introduced probes that have themselves acid-base properties can modify the compartments’ pH.

### 2.2 Phluorins

The Green Fluorescent Protein (GFP) from *Aequorea Victoria* displays an elegant autocatalytic mechanism of intrinsic fluorophore formation that involves the sequential cyclization and oxidation of a triad of Tyrosine-Serine-Glycine residues (Barondeau et al., 2003). Rothman and collaborators have generated pH sensitive GFPs by an ingenious combination of site-directed and random mutagenesis close to this aminoacid triad in the protein structure (Miesenbock et al., 1998). Following this work that gave birth to the first ratiometric and superecliptic pH sensitive GFPs termed pHluorins, many versions have been developed, to both increase their fluorescence or shift their pH response to be able to measure pH in more oxidative (mitochondrial matrix) or acidic compartments, such as TGN for example (Llopis et al., 1998; Mahon, 2011). Another important advantage is that cDNAs encoding these phluorins can be adequately fused in frame with those encoding various proteins, to target them into a particular cellular compartment or location. In this respect, a recent work describes the construction of a Lamp-1 fused pHluorin that targets mostly lysosomal compartments and enables to measure luminal lysosomal pH in good accordance with data from the previous literature (Ponsford et al., 2021). In different cell lines, this pHfluorin does neither affect lysosomal structure nor function, and has the advantage of being endogenously expressed, making a large set of functional measurements possible without any further treatment.

### 2.3 Nanosensors

A novel category of pH probes is emerging from biosensor nanotechnologies, and we will give a few examples among many. As a first example, carbon nanodots are much smaller than classical nanosensors and are therefore small enough (5–10 nm) to directly cross membranes instead of being endocytosed and accumulate in intracellular compartments. These nanodots do not exhibit detectable cellular toxicity and can be bound to various molecules, such as fluorescein or rhodamine derivatives or both. This can lead to interesting probes with ratiometric properties over a wide range of pH values (5.2–8.5), making them quite versatile tools to measure cytosolic pH (Shi et al., 2012).

Concerning lysosomal pH, Yue and collaborators (Yue et al., 2021) developed an interesting FRET-based approach, in which they used a split motif design with tetrahedral DNA that changes conformation with pH to build a nanosensor that has a very good signal to noise ratio around pH 6, well in the range of endo-lysosomal pH values. The large advantage is that such nanoparticular probes can be endocytosed and arrive in lysosomes and endosomes where they will be able to change their emission properties as a function of the pH. Taken together, those novel approaches, which take advantage of the fast development of nanotechnologies represent a future area worth developing in the field of intracellular and organellar pH measurements.

### 2.4 Intracellular Microelectrodes/Nanotubes

pH selective microelectrodes have been used for intracellular pH measurements, but as they can cause membrane damage and cytosolic leakage that will impair proper intracellular pH measurements, their use has been rather limited to large cellular systems like *Xenopus* oocytes. Much smaller electrochemical intracellular pH sensors such as viral-coat proteins-DNA nanotubes modified gold electrodes have been designed. These electrodes take the advantage of the viral coat protein from Cowpea Chlorotic Mottle Virus (CCMV) self-assembly into nanotubes when incubated with double stranded DNA. The resulting nano electrodes are then adsorbed with methylene blue, a compound whose oxidation and reduction peaks shift with pH. This change can be detected electrically resulting in nanoelectrodes that have a large sensitivity from pH 4 to 9 (Ning et al., 2014). In the experimental device, those Nanotubes are grown on DNA coated on a gold sensor plate. The viral protein-based electrodes can then spontaneously insert into cell seeded on the plate without causing damage and be used to record pH changes.

### 2.5 Limitations and Constraints

pH measurements can also be limited by the systems that lay before and after the chemical and physical properties of the probes or nanodevices used for the measurement itself. These different drawbacks can be related to the spatiotemporal features of the acquisition systems. In particular, for all measurements using fluorescence, working at very high rates and magnification automatically decreases the number of photons that can be gathered. As probe photobleaching and cell damage limit the possibilities to increase the illumination intensities, extremely sensitive cameras are then needed to overcome this problem. Similarly measuring fast kinetics of pH responses triggered by transient perturbations requires fast perfusion systems in order not to miss precious information such as the occurrence of overshoots. In our group, we could observe that such interesting signals, which were very difficult to observe using classical commercial perfusion systems, were revealed under fast laminar flow using microfluidic perfusion systems (Tran, Noblin, Bouret and Counillon, in preparation).

## 3 Summary of Cellular Acid-Base Chemistry

### 3.1 Intracellular Buffers

Solution chemistry defines the rates and equilibriums of reactions as functions of the free concentrations of reactants and products. In complex chemical systems such as cells, the situation is far from this simplified view because many species are not in their free form and are also intermediates in large reactions networks, making their kinetics dictated by multiple steps. H^+^ ions whose small size and reactivity make them capable to combine with nearly all biological molecules are archetypal of this situation.

Indeed, most H^+^ ions are combined to a bulk of biological molecules such as proteins, nucleic acids, lipids or smaller molecules, that simply carry protonable groups such as carboxylic acids, amines or phosphates (possibly free as inorganic phosphate and/or pyrophosphate; or associated with larger molecules by phosphodiester bonds). All those can operate as intracellular buffers with pK_as_ that extend over a range of 4–10, far from actual cytosolic pH values that are slightly above 7. However, as cells are extremely crowded with macromolecules, those are quantitatively important in the net free H^+^ balance. Furthermore, as protons constantly bind and unbind from different macromolecular complexes, this strongly reduces their diffusion rates, potentially leading to compartmentalization (Swietach et al., 2005).

### 3.2 Bicarbonate

**FIGURE 1:**
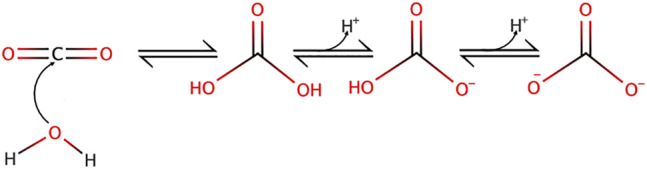
Carbon dioxide nucleophilic hydration reaction yielding carbonic acid and its subsequent dissociation into bicarbonate and carbonate ions.

The importance of the carbonic acid-bicarbonate equilibrium in setting up free proton concentration has been very nicely explained by several colleagues in excellent articles (see for example (Occhipinti and Boron, 2015)) and thus the aim here is not to go over this topic as much in too much detail but to recall the main features of this buffer. Briefly CO_2_ nucleophilic attack by water yields carbonic acid as depicted in Figure 1.

**FIGURE 2:**
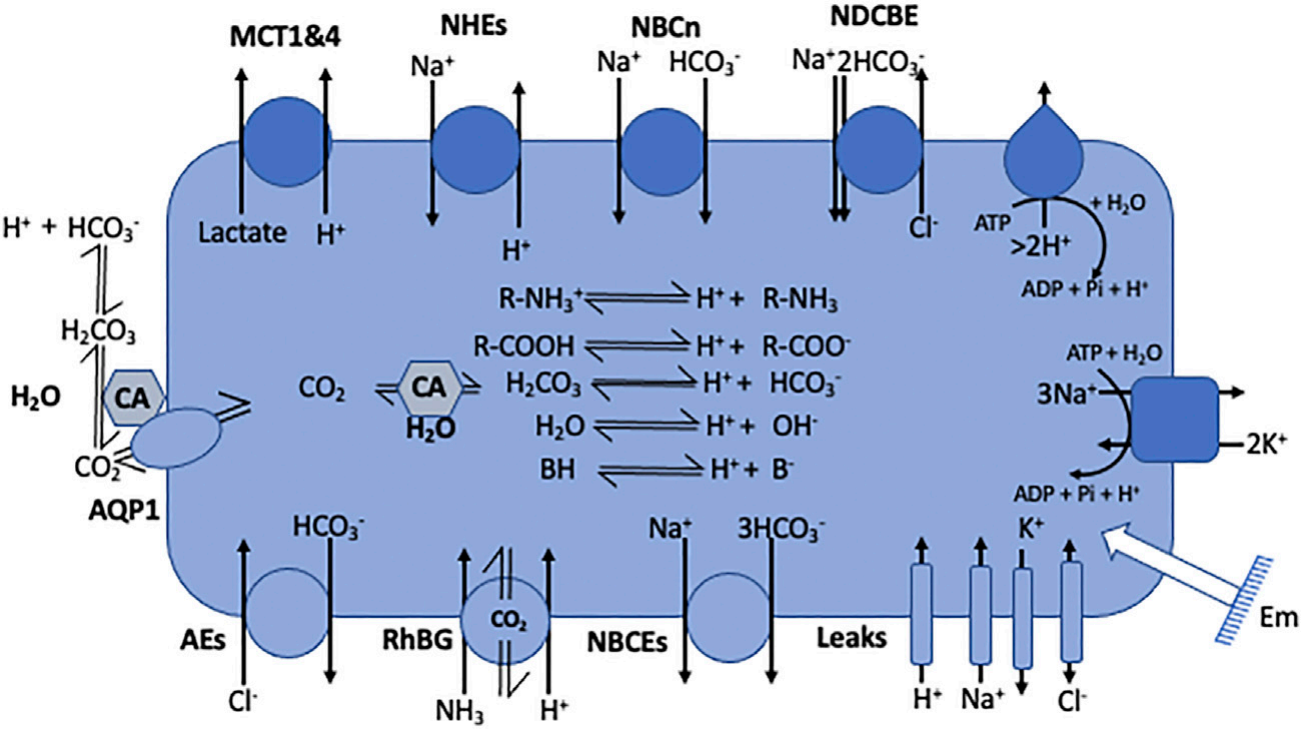
Cell model representing the main families of pH regulating transport mechanisms as listed in Table 1. The model also encompasses the electrogenic pumps and the passive channels that set the membrane potential (Em) and also use the same substrates as the coupling anions and cations that energize some of the pH regulatory transporters. It also represents the inner acid base chemistry that consumes or generates the transported acid-base equivalents.

Noticeably, the two protonation equilibria pK_as_ are 6.35 and 10.33 respectively, but those are displaced by the CO_2_ hydration reaction (Keq = 2.5 10^−3^ at 25°C) (England et al., 2011), making it an excellent biological buffer, which also couples cellular respiration to pH regulation. Bicarbonate has also been selected by evolution as one of the most important biological buffers, in a large part because it solves two main problems at the same time. Firstly, bicarbonate can bridge the gap between the too low and too high pK_a_ values of acid-base groups of the previously cited biological macromolecules. Secondly, as a gas CO_2_ can diffuse very fast in the cytosol, as well as through membranes, this diffusion being facilitated through the structures of several transmembrane proteins (Michenkova et al., 2021). Hence, CO_2_ can be viewed a convenient alternative for fast H^+^ transport in cells but also across membranes. When hydrated, CO_2_ becomes in contrast a classical acid base system that can release or capture protons, diffuse fast in the cytosol but not diffuse freely anymore across membranes. Kinetically and spatially, CO_2_ hydration is controlled by multiple isoforms of carbonic anhydrases that are exceptionally efficient enzymes and are specifically expressed extra or intracellularly. Based on their location and the existing gradients, these enzymes are thus instrumental to control CO_2_ traffic across membranes and consequently H^+^ fluxes (Figure 2).

### 3.3 Water Molecules

The equilibrium of water dissociation into H^+^ and OH^−^ ions is extremely in favor of the H_2_O molecule. In pure water at pH 7, the ratio of H_2_O over its dissociated forms is superior to 550 millions. As previously stated, cells are also crowded with acid base functions. In these conditions, it would be tempting to consider that water dissociation/association is marginal in cell physiology. This has to be however taken with caution when considering cytosolic pH for at least two reasons: 1) as water is the biological solvent, H_2_O concentration is > 55 molesl^−1^ which is orders of magnitude above the other biological molecules and 2) cytosolic pH is close to 7, which is the value at which water dissociation/association is the most labile.

## 4 Membrane Transport

As mentioned in the introduction, several reviews provide a high level of detail on membrane transporters involved in pH regulation [see for example (Casey et al., 2010; Parker and Boron, 2013; Pedersen and Counillon, 2019)]. Hence, the aim of the present section is 1) to give a short summary on these transporters in order 2) to select relevant questions pertaining to cellular acid-base physiology and 3) to illustrate how mathematical modeling can provide here interesting clues.

### 4.1 Cells are not Chemical Reactors and Require Membrane Transport

Metabolism enables cells to extract free energy from the oxidation of a large set of macromolecules. This free energy that is carried mostly by ATP is transferred 1) to coupled chemical reactions, 2) to the creation and maintenance of membrane gradients and 3) to energize molecular motors. A significant fraction of the entropy that accompanies this free energy harvest and utilization is materialized by the generation of H^+^ ions which have much more degrees of freedom than hydrogen atoms bound to large molecules. Furthermore, in all eukaryotic cells, the negative intracellular membrane potential also creates an inward proton gradient that would produce an acidic cytoplasm if not counterbalanced by opposite H^+^ membrane transport. Conversely, if not maintained also by active pumping by V-ATPases, the acidic pH of intracellular compartment should also dissipate and acidify the cytosol.

Taken together, to maintain a steady-state pH, eukaryotic cells need to divert a significant fraction of their energy to maintain a high level of H^+^ transport across their plasma membrane. This is achieved either directly through proton transport or indirectly through the transport of acidobasic molecules that can carry H^+^ or buffer capacity. Strikingly, we can observe that membrane transport roughly involves the same chemical moieties as those previously discussed, namely H^+^, carboxylates, ammonium, or bicarbonate. Furthermore, as most of these transporters carry ionized species, their activity is directly or indirectly linked to ion fluxes and membrane potential and therefore to the cellular overall electrical activity.

### 4.2 More Than 63 Genes Encode pH Regulatory Plasma Membrane Proteins

A comprehensive list of transporter families in humans can be found on several sites for example https://www.guidetopharmacology.org (channels and transporters subfamilies) (IUPHAR/BPS). These transporters whose functions are instrumental for pH regulation are summarized in Figure 2 and the corresponding gene nomenclature and gene numbers are given in Table 1.

**TABLE 1:** Compilation of the proteins whose main physiological function is to transport H^+^ or acid base equivalents across the plasma membrane to regulate pH. Source: https://www.guidetopharmacology.org.

Type of Protection	Function in PH regulation	Nomenclature	Number of genes
H^+^V-ATPase	H^+^ translocation subunits	V-ATPase V0 Subunit, V-ATPase V1 Subunit	10, 13
ATP6V0&1	ATP tumover subunits	V-Atpase V1 Subunit	4, 2, 2, 1
Bicarbonate Transporters SLC4 and SLC26	Electroneutral Cl-/HCO_3_-Exchange, Eletroneutral Cl-/HCO_3_-Exchange, Eletroneutral NA^+^/HCO_3_-Cotransport, Na+- Driven Cl-/HCO_3_-Exchange	AE1-4, NBcn1and 2, NBce1 and 2, NDCBE	9, 4
NA^+^/H^+^Exchangers SLC9a,b and c	Eletroneutral NHEs Atypical NHA (possibly eletrogenic)	NHE1 to NHE9, SLC9b and C	4
Monocarboxylate Transporters SLC16	Lactate coupled proton transport	MCT1 TO 4	1, 1
NH_3_/H^+^ Cotransporters SLC42	Ammonium transport	RhBG SLC9b and C	12
Carbonic Anydrases CA1-CA12	CO_2_ hydration->indirect proton transport	Also termed car	63

This table shows that evolution has invested in about 63 genes in humans to encode proteins or their subunits that are instrumental for H^+^ or acid base equivalent transport across the plasma membrane in mammalian cells. This number could be revised upward if one considers all the genes encoding transmembrane transporters and channels that transport other vital substrates but are directly or indirectly coupled to proton transport. Those are phosphate transporters or cotransporters, (SLC20, SLC34) H^+^-coupled metal cotransporters (SLC11), H^+^ coupled aminoacid cotransporters (SLC36) or H^+^/Ca^2+^ ATPases (PMCA). Furthermore, there is now clear evidence that in their gas form, CO_2_ or NH_3_ can also cross membranes through the structures of Aquaporins (AQP1) or Rhesus proteins (Rh) that could therefore be considered as involved in pH regulation (Michenkova et al., 2021). There is also a set of transporters and pumps that establish an acidic pH in the lumen of intracellular compartments and to this end take up protons from the cytosol. However, their steady-state contribution is challenging to quantify because of the multiplicity of different compartments, which all maintain a specific pH, and because of their different turnover rates in vesicular trafficking. For the sake of simplicity, we will from now only consider the plasma membrane proteins that take part in pH regulation.

While several structures had been solved by X ray crystallography, thanks to cryo-Electron Microscopy, many structures that remained unknown for many decades since the cloning of their cDNAs, are now available. Moreover, resolutions are generally sufficient to identify important functional sites for substrate and inhibitor interaction and to locate the functionally important residues previously identified by mutagenesis.

The interested readers can find the cryoEM structures of the Sodium-driven Chloride/Bicarbonate Exchanger NDCBE (SLC4A8) in (Wang et al., 2021), of the electrogenic sodium-bicarbonate cotransporter structure in (Huynh et al., 2018), and of NHE1 and nine structures in (Winklemann et al., 2020; Dong et al., 2021). Other important structures have been obtained by X-ray diffraction, for AE1 in (Arakawa et al., 2015), and for monocarboxylate transporters (Bosshart et al., 2019; Zhang et al., 2020). Interestingly the 3D structures show a similar folding for the coupled transporters with a dimeric organization that opens possibilities for cooperativity and multiple allosteric regulations. Composite images have also been obtained for V-ATPases (Abbas et al., 2020).

## 5 Addressing the Redundancy of pH Regulation

Taken together, this rather large number of genes encoding pH membrane regulators questions the significance of such a redundancy as established through natural selection. In part, this can of course be explained by the fact that different cell types need different equipment for transport. In this context, gene duplication during evolution could have selected specialized isoforms whose expression would boost a particular function in a given tissue. For example, the kidney tubule uses a combination of apical H^+^-ATPase and Na^+^/H^+^ exchangers NHE2 and NHE3 to secrete H^+^ ions in order to reabsorb bicarbonate from the tubule lumen, while they express the housekeeping Na^+^/H^+^ exchanger NHE1 basolaterally for pH regulation (Wang et al., 2001). Similarly, in the small intestine, NHE2 and NHE3 are expressed apically where they mediate Na+ and water intestinal absorption as well as luminal acidification. In parallel those exchangers have supplementary functions in establishing a pH gradient for divalent metal transport as well as in regulating the intestinal microbiome composition and properties through their action on luminal pH. In parallel, the basolateral NHE1 ensures housekeeping pH regulation (Pedersen and Counillon 2019).

However, this does not fully account for more subtle redundancies. In particular, different molecular machineries having the same overall function for pH regulation, such as NHEs, NBCs, monocarboxylate transporters and in some cases V-ATPases can be expressed in the same cells. Understanding why evolution has selected the expression of such different genes instead of relying on a single robust transporter is nontrivial because redundancy has a cost, which implies that the advantage must be immediate to be selected. This is contrast to human engineering where redundancy can be introduced to prevent future failures that may or may not happen. Beside the evolutionary aspect of this question, documented answers may provide clues to better understand in which pathological situations, e.g. ischemia-reperfusion or cancer, such a redundancy could be functionnally beneficial or detrimental.

### 5.1 Modeling Addresses the Minimal Set of Membrane Transporters for Acid-Base Regulation

To answer the present question, a possible approach would be to identify the minimal set of transmembrane transporters that would be required to maintain both the steady-state pH of a very simple cellular system close to neutrality and to restore it when subjected to transient and/or persistent perturbations. We and others (Swietach et al., 2005; Occhipinti and Boron, 2015; Counillon et al., 2016) resorted to mathematical modeling to establish cellular systems in which pH regulation by membrane transporters could be simulated adequately. As previously mentioned, it is mandatory to plug in minimal cytosolic chemistry in the form of buffers, bicarbonate and water as well as all membrane potentials and Na^+^, K^+^ and Cl^−^ fluxes through channels that are indirectly coupled to H^+^ transport because they will affect the gradients of cotransporters’ counterions.

**FIGURE 3:**
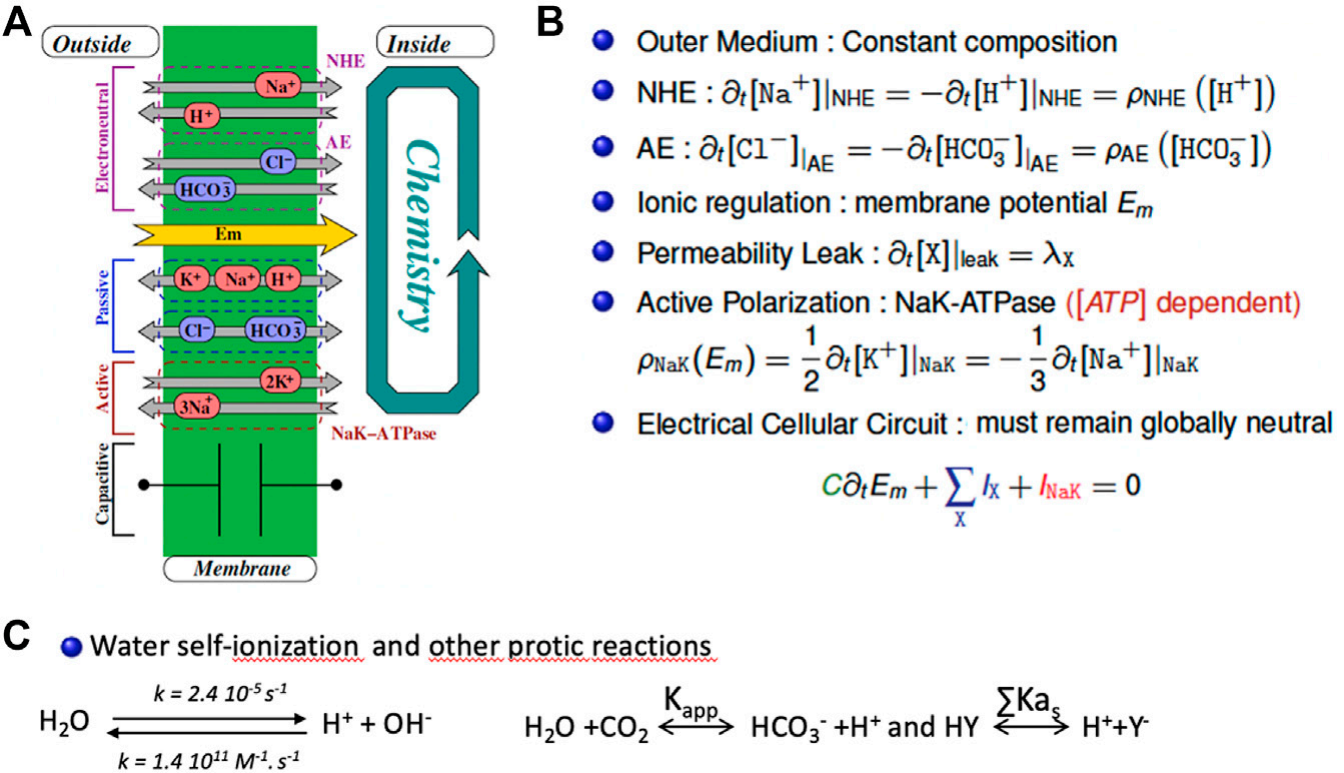
The mathematical bases of the minimal model for intracellular pH regulation. **(A)** Schematic representation of the cellular system encompassing a membrane pump (Na-K ATPase), leaks, an alkalinizer (NHE) and an acidifier (AE). **(B)** the corresponding set of coupled differential equations that mathematically depict the system that include the different stoichiometric coefficients. Detailed rate expressions (the ρ terms) are given in (Bouret et al., 2014) **(C)** The fast-relaxing equilibria for water, bicarbonate and buffers.

Based on this rationale and on equations built from experimental data for ion transporters and channels, we built a fully tractable model with only two antagonistic housekeeping transporters possessing the kinetic features of NHE1 and AE2. Figure 3 shows the correspondence between the set of transporters, channels and equilibria present in this minimal system (A) the resulting differential terms (B) and fast-relaxing equilibria (C). Note that the same ion (e.g., Na^+,^ or HCO_3_^−^) can appear at multiple places in (B) and/or (C), reflecting the fact that multiple transporters, channels and/or equilibria share the same substrates. We account for NHE and its molar rate ρ_NHE_ exchanging one outer Na^+^ with one inner H^+^. Likewise, the Anionic Exchanger AE transports a bicarbonate anion outside of the cell whilst bringing a chloride anion inside at a molar rate ρ_AE_. Those two exchangers are electroneutral. In parallel, a membrane potential E_m_ is mainly developed by the electrogenic NaK-ATPase that intakes two K^+^ and expels three Na^+^, (with a molar rate ρ_NaK_), hence maintaining a negative inner voltage. Furthermore, any species X may flow through membrane channels or their equivalents according to its passive electroosmotic gradient and producing a leak λ_X_ that depends on the permeability of the concerned species. Finally, the membrane potential shall vary to balance the net charge transport with the help of the membrane capacitance. As this approach encompasses all protic reactions, as well as matter and charge conservation, it significantly departs from earlier pH regulation models that only partly take these different variables into account and have for this reason been questioned elsewhere (Kurtz et al., 2008; Nguyen et al., 2011).

The interested reader can find the detailed kinetic terms included in the ρ molar rates present in each individual differential equation in (Bouret et al., 2014). Consequently, we model the cellular system as a set of coupled chemical reactions that put together both the transport mechanisms as well as the cellular chemistry that includes water, bicarbonate, and buffers (Figure 3C). Consequently, the whole model for the cellular inner chemistry can be considered as a set of N chemical reactions (involving at least N species). Each reaction may evolve with its chemical extent to reach its equilibria. Accordingly, starting from a given set of concentrations, we reach N simultaneous equilibria depending on N independent extents. To compute them we use a non-linear, zero finding Newton’s algorithm, modified to ensure the positivity of the resulting concentrations (that we call the Newton^+^ algorithm).

Let us now impose a perturbation, namely an intake of some species, to these equilibria: under the assumption that the relaxation times of each reaction is fast compared to the transport rates. The system will produce a set of extents that will bring each reaction back to the next allowed equilibrium. From a theoretical point of view, this method is the way to find the evolution of coupled concentrations subject to “slow” perturbations under N algebraic, chemical, and “fast” constraints, and requires a fair amount of algebra as described in our previous work. From an experimental point of view, the water self-ionization constants yield a relaxation time in the microsecond domain for the physiological pH ranges, and, according to literature, we expect the protic reactions to relax with a characteristic time around or below a few milliseconds for the same conditions. Therefore, our approximation is quite correct since any of the used transports requires at least a few seconds to carry out a significant change in cytosolic concentrations. In order to numerically integrate the differential system that we derived, we designed a home-made C++ software, based on and adaptive Runge-Kutta solver which we modified with 1) the effect of the chemical, algebraic constraints and 2) our Newton^+^ algorithm, to ensure that every trial set of concentrations respects the set of equilibria.

Such a simplified model is able to fully recapitulate intracellular pH regulation (Bouret et al., 2014; Counillon et al., 2016). This model predicts a large and robust area of intracellular pH in the range of biological values and a powerful ability to compensate for transient or sustained perturbations. The corresponding simulations are very similar to curves obtained both in our experiments and in the literature, using different techniques to measure intracellular pH, as described in the first section of this manuscript.

Taken together we can conclude from this model that only one acid loader and one acid extruder working antagonistically are sufficient for an accurate pH regulation at physiological values. Such a result is striking as it comes in apparent contradiction with our previous analysis from genomes that natural selection has built a large set of redundant transporters. Although one could always argue that a mathematical model cannot encompass all the aspects of a real-life cell, the efficiency of pH regulation found for such a minimal set of effectors seems nevertheless too robust to be an artifact of calculation. It is also supported by the following experimental evidence. In pioneer experiments performed several decades ago, it has been possible to select or build NHE deficient fibroblasts, that can survive and grow perfectly well in cell culture conditions (Pouyssegur et al., 1984), and recover from acute acid challenges in the presence of Na^+^ and bicarbonate as they express NBCn1. Conversely, the same cells re-expressing NHE1 can fully resist an intracellular acidification in the absence of bicarbonate, where NBCn1 is inactive, provided that Na^+^ is present to energize NHE1 (Franchi et al., 1986). Taken together, we can conclude that coupling one strong acid loader with one strong extruder is sufficient to fully compensate for acute intracellular pH variations. Ergo, the reasons nature has chosen to co-express multiple transporters have to be found from a deeper analysis. This will be performed in the next chapters in which we will add functional redundancy to our model.

### 5.2 Modeling Functional Redundancy

#### 5.2.1 Sustained Acidosis

**FIGURE 4:**
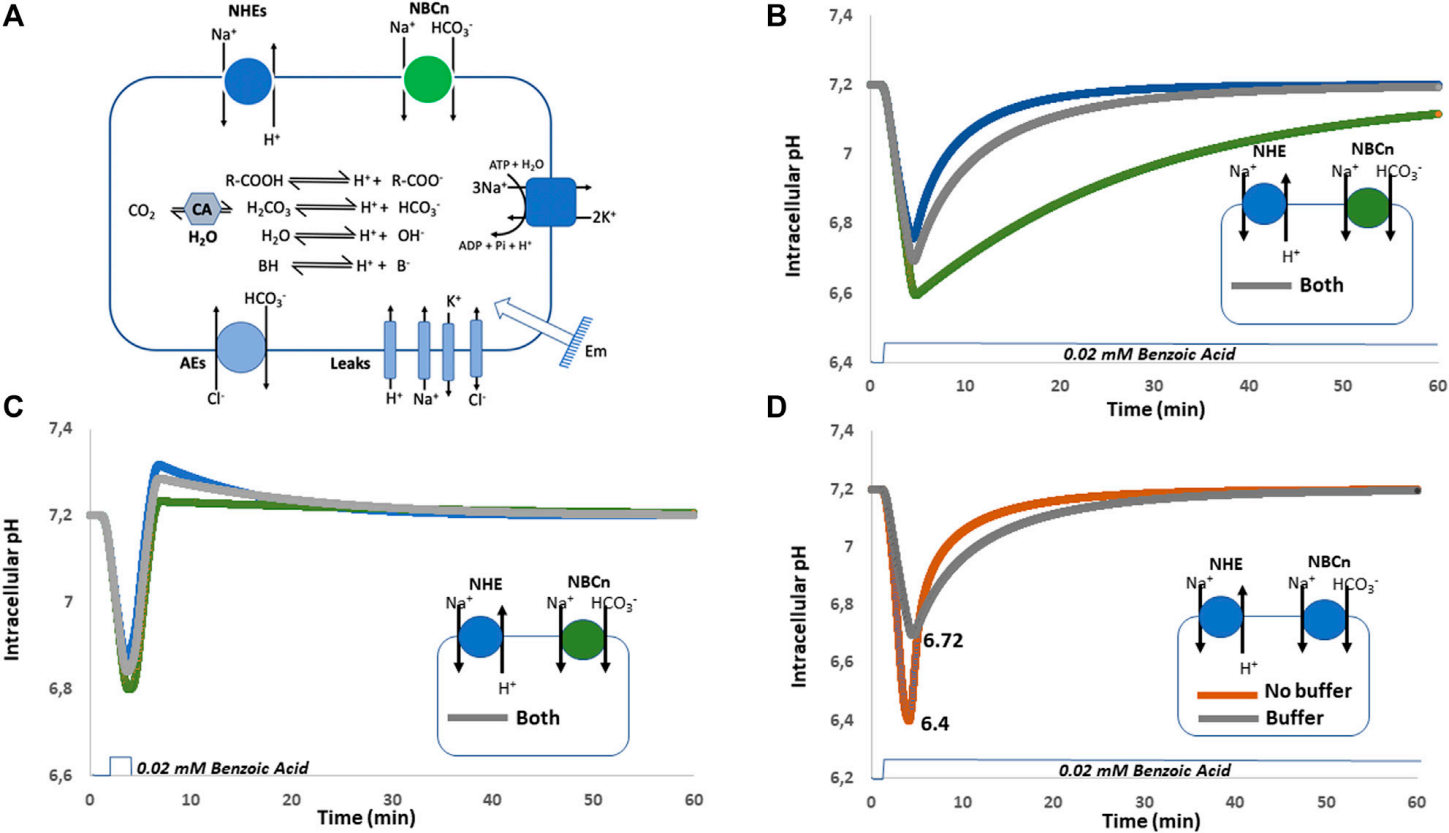
Simulations of forced sustained or transient acidosis by weak acid diffusion in cellular models expressing either NHE1, NBCn1 or both, in the presence or absence of buffers. **(A)** Cellular system used for the simulations shown in **(B–D)**. **(B)** Sustained acidification induced by 20 mM weak acid with only NHE1 (blue), NBCn1(green) or both (grey). **(C)** Transient acidification induced by 20 mM weak acid with the expression of NHE1 (blue), or NBCn1 (green) or both (grey) in the presence of intracellular buffer. Note the strong overshoot that takes place with NHE1 only and how it is damped by the coexpression of NBCn1. **(D)** Sustained acidification induced by 20 mM weak acid with the expression of both NHE1 and NBCn1 in the absence (brown) or in the presence (grey) of intracellular buffering. Note the differences in the extent of acidification and kinetic of pH recovery.

We constructed a cellular model (Figure 4A) containing the previous minimal ion transport and pH regulating equipment in which we simulated the expression of NHE1, NBCn1, which work both at alkalinizing cells, separately or together. In this later condition we were careful that the sum of their activities triggers the same Na^+^ flux as the NHE1 or NBCn1 only simulations. This is mandatory to make all simulations comparable because if we simply add NHE1 and NBCn1 at the same levels as alone, this will double the Na^+^ loaders’ quantity and automatically change the Na^+^ fluxes and ionic steady state values of the simulated cells. This will affect all ion and pH regulation in a pleiotropic manner and has to be avoided to make tractable comparisons. Another obvious advantage of the modeling approach is that we can also simulate the absence of cellular buffers, which is biologically impossible as those are in a large part constituted by cellular macromolecules. Figure 4B shows a simulation of pH recovery following a sustained acidosis triggered by the addition of 20 mM of a permeant weak acid possessing a pK_a_ of 4.2 such as benzoic acid, either in cells expressing only NHE1, (blue curve), only NBCn1 (green curve), or both (grey curve). When alone, both NHE1 and NBCn1 can compensate for the acidification, but with a much lower rate for NBCn1, whose effect on H^+^ is indirect and slowed down by the coupling with bicarbonate chemistry. When the two transporters are coexpressed, NHE1 seems to dominate the pH recovery as the slower NBCn1 minimally affects the rate of pH recovery. This result is consistent with the previous finding that NHE1 alone is fully able to restore a physiological pH, but again this questions the advantage of redundant NBCn1 expression, which does not seem noticeable when simulating such a sustainable acidification. As the two transporters are coexpressed in many cell types, this indicates again that we need to further explore a wider range of cellular responses to pH changes to find the clues to this apparent paradox.

#### 5.2.2 Transient pH Changes and Overshoots

As displayed in Figure 4C, we now perform a simulation of pH recovery, using the same system with NHE1 only, (Blue curve), NBCn1 only (Green Curve), or both (Grey Curve). The important difference is that the simulated acidosis triggered by the addition of 20 mM of benzoic acid is transient instead of continuous. As previously, the pH recovery is the swiftest in NHE1 only expressing cells but it can be observed that the rapid release of the acidosis triggers here a strong overshoot towards alkaline pH values. In the presence of NBCn1 only, pH drops slightly lower, recovers more slowly but with a negligible overshoot, here also due to coupling to bicarbonate chemistry. Interestingly when both transporters are present, NHE1 overshoot is significantly reduced by NBCn1.

Taken together, these simulations show that the selective advantages of functional redundancy are consistent but rather subtle. Combining a fast H^+^-transporting exchanger with a slower bicarbonate cotransporter preserves the ability to both compensate sustained and transient acidifications and, in the meantime, allows to damp strong overshoots that produce potentially damaging alkalinization and intracellular Na^+^ overload. This emerging picture constitutes, in our opinion, an area worth of experimental investigation to pursue a deep understanding of this strong redundancy of transporters and channels that regulate physical parameters.

#### 5.2.3 The Interesting Role of Buffers

The simulations in Figure 4D illustrate that intracellular buffers couple intracellular chemistry with ion transport in a subtle way. Without buffers, pH drops to a minimal value of 6.4 and then very swiftly recovers due to the conjugated activities of NHE1 and NBCn1. In contrast with intracellular buffers, the lowest pH reached in the same conditions is 6.72, but the recovery itself is slower as it is also damped by the presence of buffers. This longer recovery prolongs the activity period of the above-mentioned transporters and even though pH does not drop as low, this nevertheless increases the transporters’ activity resulting in a larger Na^+^ load (by about 20%) and counterions’ perturbation. Taken together, this simulation clearly shows that, as buffers protect cells against falling in a lethally low pH area, an interesting consequence is that this occurs at the expense of the recovery rate and engenders greater ionic perturbations that only can be resolved at a metabolic cost.

## 6 Concluding Remarks

Taken together studying intracellular pH regulation is both essential and complex. Its measurements that have been pioneered more than 4 decades ago have led to robust and numerous results that paved the way to cloning the genes encoding pH regulatory transporters. The field is experiencing a second birth with the advent of measurement techniques that should enable both greater space resolution and dynamic range. This is particularly important for the study of pH regulation in intracellular compartments, that have always been more challenging because of their small size, rapid movements, multiplicity and by their difficult accessibility inside cells. As previously mentioned, while improving pH quantitation itself is mandatory, it has to be accompanied by parallel development of the experimental setups, namely acquisition and perfusion, and a calibration whose resolution must be in line with the probes’ performances.

Another important point is that pH dynamics cannot be simply described as a combination of the transporters’ rates because their activity is coupled to the bulk of all cellular ion channels, active and passive transporters, even if those do not directly act on pH. Furthermore, as protic reactions are massive within a cell, it is also mandatory to take cellular chemistry fully into account as it affects the availability of the transporters’ substrates as well as the protonation and deprotonation of groups that act as buffers.

With respect to such a complex biochemistry, building mathematical and computational models can yield valuable insights. For this, they must be based as much as possible on experimentally determined equations of transport and it is mandatory that they fully respect physics and chemistry, notably charge and matter conservation. As shown in this article, this enables to build a minimal intracellular pH regulation and provide subtle insights into fundamental biological questions pertaining to functional redundancy. The resulting simulations indicate that redundancy ensures a tradeoff between safety, kinetics and transient overshoots. Whether the respective gene expression patterns have been shaped by evolution to fully optimize this tradeoff is a fundamental biological question worth investigating. The ease by which it is now possible to perform gene editing in combination with the previously described improvements in measurement techniques opens an area worth investigating to experimentally challenge this output of mathematical modeling.
